# *Lebbiea* (Podostemaceae-Podostemoideae), a new, nearly extinct genus with foliose tepals, in Sierra Leone

**DOI:** 10.1371/journal.pone.0203603

**Published:** 2018-10-05

**Authors:** Martin Cheek, Aiah Lebbie

**Affiliations:** 1 Science, Royal Botanic Gardens, Kew, Richmond, Surrey, United Kingdom; 2 National Herbarium of Sierra Leone, Department of Biological Sciences, Njala University, Sierra Leone; Universitat Bayreuth, GERMANY

## Abstract

*Lebbiea grandiflora* (Podostemaceae), a rheophytic herb from the Sewa River rapids in Sierra Leone, is described as a new species. It is the first new African genus of Podostemaceae published for 30 years. First collected in May 2017, the species is assessed as Critically Endangered using the IUCN 2012 standard. It is on the edge of extinction with a small population at a single site threatened by deposition of gravel and sand from alluvial gold and diamond mining upstream, and a planned hydro-electric dam. The new genus is unique in Podostemaceae in a) its highly developed and robust style-stigma structure in which the bases of the two style-stigmas unite to form a bifurcate funneliform-cylindrical structure, with a reflexed, blade-like apex that extends half-way around the perimeter of the ovary-fruit towards the base of the ovary-fruit, b) a specialised andropodium, with robust, self-supporting capacity, having differentiated thickened central, and angled, thinner marginal areas (in other Podostemaceae the andropodial structures are undifferentiated), c) the pillar-like haptera which completely elevate the crustose root above the substrate. *Lebbiea* is placed in Podostemoideae, necessitating amplification of the delimitation of that subfamily in which it is additionally unique in having the foliose tepals characteristic of the basal subfamilies Weddellinoideae and Tristichoideae.

## Introduction

Podostemaceae are a pantropical family of annual or perennial herbs placed in Malpighiales in a sister relationship with Hypericaceae [1]. Some 300 species are accepted globally, in c. 54 genera [2]. Species numbers are highest in tropical America, followed by Asia, with Africa having c. 106 species. All species of the family are restricted to rocks in rapids and waterfalls of clear-water rivers, and are therefore rheophytes. However, this very habitat is being increasingly impacted by human activities and exploited for hydropower at risk to the survival of the Podostemaceae they contain [2–7].

Three subfamilies are recognised in Podostemaceae: the pantropical Tristichoideae with 3 genera, 10 species, the monotypic New World Weddellinioideae, and, with the majority of genera and species, the pantropical Podostemoideae. The Podostemoideae have only vestigial, filiform tepals, and have a specialised, balloon-like structure, the spathellum, which protects the developing flower. Tristichoideae and Weddellinioideae, sister to Podostemoideae, lack a spathellum and have well-developed, protective tepals that protect the developing flower [2,8].

All African species fall in subfamily Podostemoideae (`Podostemoids’) apart from one, *Tristicha trifaria* (Willd.) Spreng. (Tristichoideae). Most of the African species of Podostemaceae-Podostemoideae are narrow endemics, many being species known from only a single waterfall and often restricted to single river systems. New discoveries of species are still being made frequently [3–7, 9–15]. In Africa, Podostemoideae occur only south of the Sahara, mainly in the equatorial belt, with only one species reaching S. Africa. Diversity is low in the congolian forest and eastern African areas. Known generic and species diversity is focussed in southern Cameroon.

Important characters in defining genera in Podostemaceae-Podostemoideae are the position of the flower in the unruptured spathellum, and the shape, locule number, and sculpture of the ovary. At the species level, important characters are the shape and relative proportions of spathellae, stigmas, anthers, filaments, gynophores, pedicels, and leaves.

The current generic classification of African Podostemaceae is based on the framework established by Cusset [16–22]. This work was compiled and updated by Rutishauser et al. [23] who recognised c. 85 species in 16 genera. With new discoveries and extensive field research now led by resident African botanists, the numbers have increased to 106 species to date, and are likely to increase further.

During a botanical survey to inform an Environmental Impact Assessment for a proposed hydroelectric project, among other new taxa discovered [24], *Ledermanniella yiben* Cheek (Podostemaceae) was discovered seemingly globally unique to the designated reservoir area behind the proposed dam at Bumbuna-Yiben [6]. Therefore, it was decided to widen geographically the survey for Podostemaceae in Sierra Leone with the object of discovering additional sites for *Ledermanniella yiben* so that it could be shown that its loss at the type locality at Yiben would not result in its global extinction. Accordingly, the second author led a team of six technicians and local community representatives to execute this object in April-May 2017. Among the specimens of Podostemaceae collected was *Lebbie* A2721, with several characters previously unknown in the family. Under this specimen number, material was collected representing numerous individiuals, all of which were uniform in their morphology. In this paper, the specimen is described as a new genus and species to science, and we discuss its subfamilial placement and generic affinities.

## Materials and methods

### Ethics statement

*Lebbie* A2721, the herbarium specimen which triggered this paper, was collected during follow-up field studies for an Environmental Impact assessment of the Yiben hydroelectric dam. This was part of a survey co-ordinated and managed by the international environmental consultancy company The Biodiversity Consultancy (TBC) for the engineering company Joule Africa.

The National Herbarium of Sierra Leone, Njala University, which conducted the field studies, has the national statutory responsibility for study of the vegetation and plant species of Sierra Leone. As such, it requires no individual permits in order to conduct field surveys. A permit to export herbarium specimens was issued by The National Herbarium of Sierra Leone on 26 May 2017. This was supported by a phytosanitary permit from the Ministry of Agriculture, Forestry and Food Security.

The study area is in the Eastern province of Sierra Leone between Kenema District and Kono District in one of the many rapids on the Sewa River. The site is not privately owned, nor protected. The taxon represented by *Lebbie* A2721 is not currently a protected species in Sierra Leone.

### Field survey

To discover potential sites for Podostemaceae in Sierra Leone, waterfalls and rapids were detected by visually searching Google Earth imagery of Sierra Leone. Georeferences of sites were read-off and recorded. Waterfalls and rapids with road access were prioritised for attention. The georeferences were used to navigate to the waterfalls and rapids targeted. A team of six assistants were led by the second author over 26 days (17 April -11 May 2018), in the course of which 41 specimen numbers were collected. Specimens were collected of each morpho-species encountered at each site. Due to the season, all material was already dried and dead on collection. Therefore, samples were placed into paper packets numbered and cross-referenced to field data. Packets were stacked and shipped to the Royal Botanic Gardens (Kew) in a stout printer-toner cartridge box to avoid crushing the fragile material.

### Taxonomic treatment

Owing to the season of collection (late dry season), the material was already dead when collected, and so neither pollen, nor DNA could be obtained. However, in view of the rarity and threats to the survival of this taxon which may result in its immediate extinction we considered it imperative to formally name it as soon as possible so that a conservation assessment can be considered by IUCN [25]. It is to be hoped that additional and better material of this taxon might be obtained in future, but given the high likelihood of extinction, this may never be possible.

A comparative morphological study of the new taxon was conducted using collections from the following herbaria: BM, FBC, FHO, HNG, K, P, POZG, SL, WRSL. BR and COI specimens were not accessible since they were being digitised during the period of study. Codes for cited herbaria follow Index Herbariorum [26]. Cited specimens which have been seen by one or both authors are annotated “!”, prefixed by a seven-digit number for those with barcodes.

The overall morphology was documented, discussed, described and illustrated following botanical standard procedures as documented in [27]. Information about habit, habitat, and distribution was taken from specimen labels and field observations made at the time of collecting. Specimens and/or protologues and/or descriptions [8] of all Podostemaceae genera were studied and compared with the new taxon. The conservation status was evaluated using IUCN criteria, using the IUCN-preferred grid cell size of 1 km^2^ for riverine organisms [25].

Names of species and authors follow IPNI [28]. Herbarium material was examined with a Leica Wild M8 dissecting binocular microscope fitted with an eyepiece graticule measuring in units of 0.025 mm at maximum magnification. The drawing was made with the same equipment with a Leica 308700 camera lucida attachment.

The format of the description follows [6,7].

### Nomenclature

The electronic version of this article in Portable Document Format (PDF) in a work with an ISSN or ISBN will represent a published work according to the International Code of Nomenclature for algae, fungi, and plants [29], and hence the new names contained in the electronic publication of a PLOS article are effectively published under that Code from the electronic edition alone, so there is no need to provide printed copies.

In addition, the new names contained in this work have been submitted to IPNI, from where they will be made available to the Global Names Index. The IPNI LSIDs can be resolved and the associated information viewed through any standard web browser by appending the LSID contained in this publication to the prefix http://ipni.org/. The online version of this work is archived and available from the following digital repositories: PubMed Central, LOCKSS.

## Results

### Taxonomic treatment

**Lebbiea** Cheek gen. nov. [urn:lsid:ipni.org: names:77188050-1]Type: *Lebbiea grandiflora* Cheek sp.nov.

**Fig 1:**
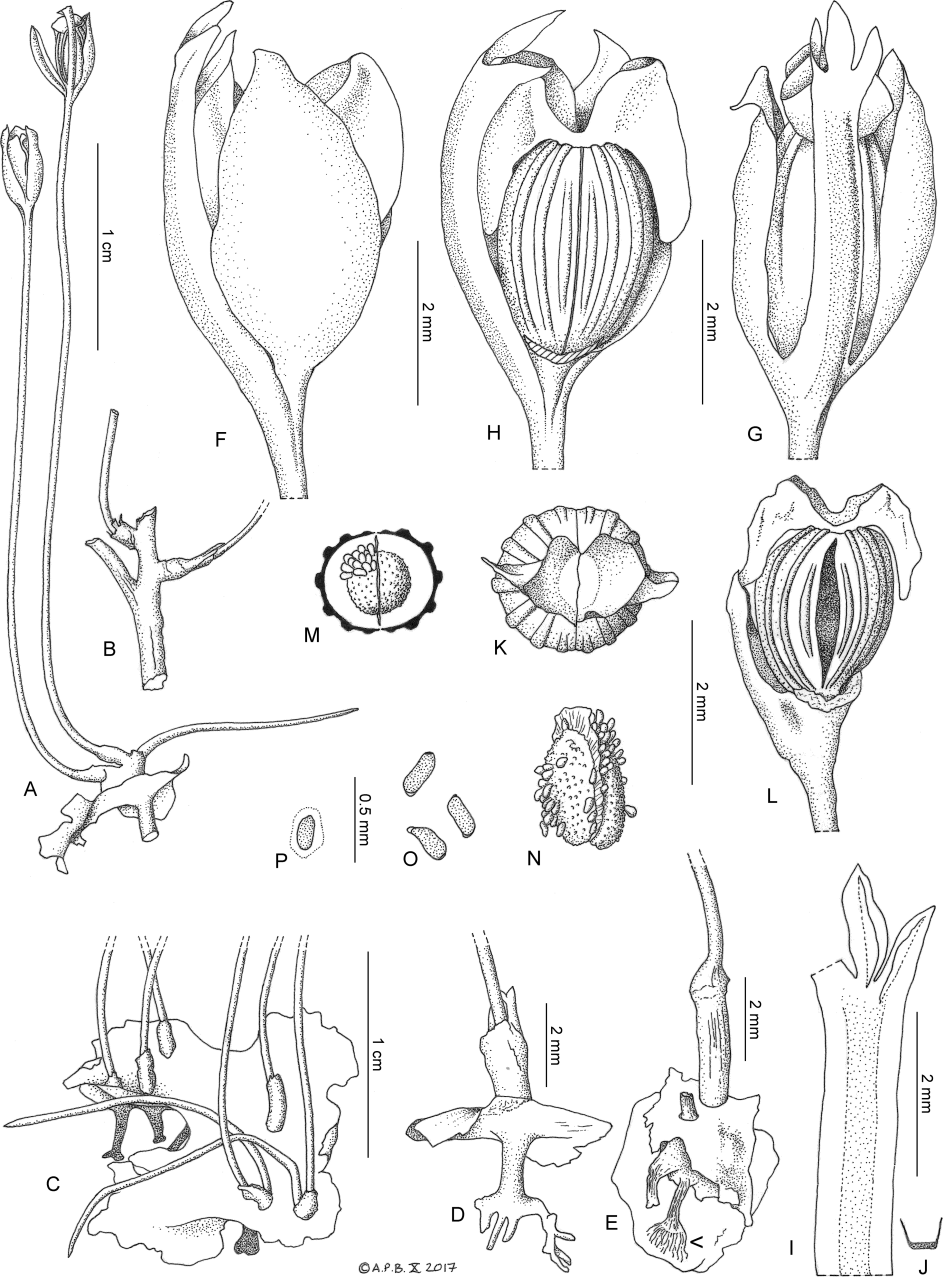
*Lebbiea grandiflora*. A. habit, whole plant, in fruit, showing the flat root, a pillar-like ‘haptera’, and a shoot with three inflorescences, B. detail of shoot with three branches, C. view of upper surface of a flattened root, with six short, erect shoots, each with 1–2 1-flowered inflorescences emerging from spathellum remains, D. side view of plant showing, on the lower surface of the flattened root, the pillar-like haptera, branched at base; upper surface of root with spathellum-sheathed inflorescence base, E. plant attached to rock by weft of thread-like root hairs (indicated with arrow) from base of pillar-like haptera; upper surface of flattened root with two shoots, F. side view of flower showing one of two tepals in full frontal view, G. as F. with tepal removed, exposing the gynoecium with, to left, the arched-over androecium, H. side view of flower with androecium in centre, two tepals flanking the gynoecium, I. androecium (leftmost of three anthers missing), J. transverse section of andropodium, K. view of gynoecium from above showing funneliform style-stigma base, L. fruit, dehisced, M. transverse section of bilocular fruit, showing septum and placentae, N. placentae with seeds, divided by septum, O. seeds, P. seed with mucilage outer layer. Drawn by Andrew Brown from *Lebbie* A2721.

Diagnosis: differing from all other Podostemaceae in the basally connate style-stigma pair which form a short, bifurcate, funneliform cylinder, the apices of each style-stigma reflexed, forming a keel which encircles the distal perimeter of the ovary (in all other species the stigmas are not divided into basal and distal parts, and if conjoined never form a cylinder, nor have a keel-like distal part); also differing from all other Podostemaceae in the robust, free-standing, concave andropodium, differentiated into thinner marginal and thickened central portions (not depending on hydrostatic pressure to stay erect, not flat or cylindric, undifferentiated); differing from all other Podostemoideae in the ovate, concave, tepals that conceal the ovary (not filiform, inconspicuous) (Fig 1)

***Lebbiea grandiflora*** Cheek sp. nov. [urn:lsid:ipni.org: 77188051-1]Type: Sierra Leone, Sewa River, between Fomaya (Kenema District) and Ngnawama (Kono District), 257 m alt., fr. 5 May 2017, *Lebbie* A2721 (holotype K! K000875049; isotypes SL!, US!, ZT!)

Annual rheophytic, glabrous, herb. Root flat, thallus-like, slightly undulating, irregular orbicular or elliptic-oblong, margin irregularly lobed, 0.3–1.5 cm diam. in plan view, 1–2 mm thick, not appearing to adhere to substrate, but to be elevated on pillar-like ‘haptera’ (Fig 1A, 1C and 1E). Haptera depending from the lower surface, 1–3 mm long, 0.3–0.8 mm diam., base sometimes branched, anchored to the substrate by numerous thread-like root-hairs (Fig 1 E). Upper surface with 1–5 erect, short sessile or long shoots. Shoots not marginal but approximately corresponding in position with the pillar-like structures on the lower surface, each 0.1–8(–14) mm long, terete, 0.3–0.75(–1.5) mm diam., unbranched or 1–4-branched (Fig 1B); internodes 0.1–2 mm long, branches usually short, 0.1–4 mm long. Leaves 1–2 per stem, erect, terete, filiform, tapering from base to apex 11–15 mm long, 0.5 mm diam., at base, base lacking sheath or stipules, apex entire, acute.

Inflorescences 1-flowered, developed inside spathellae, terminal on stem branches, or appearing sessile, arising directly from the root, on very short stems. Spathellum, after dehiscence falling (Fig 1A & 1E) or persisting (only seen post-mature, imperfectly preserved Fig 1B–1D)), cupular or shortly cylindrical, 1.5–2.5 mm by 1–1.5 mm, apex more or less truncate or bilobed; pedicel erect, 2.9–4.2 cm long, terete, (0.2–)0.3 mm diam., base thickened, c. 0.8 mm diam.

Flowers at anthesis erect, bisexual, with bilateral symmetry, 4–5 mm long x 3 mm x 2.5 mm; in bud unknown. Tepals 2, erect, concave, opposite, ovate, 4 mm by 2 mm, apex acute, base inserted immediately below the androecium and gynoecium, or the androecium inserted at the same level, or below, the tepals. Androecium inserted between the two tepals, of 3 united stamens, the filaments forming a common androecial structure, the andropodium, which arches over the gynoecium and tepals, the anthers held over one of the two stigmas. Andropodium robust, rigid, dorsiventrally flattened, concave in transverse section, 4.5–6 mm by 0.7–0.9(–1) mm, marginal third on each side membranous. Anthers 3, subequal, the central anther slightly constricted at the base, the lateral anthers not constricted, ovate-oblong or narrowly ovate (0.5–)1.2–1.3 mm by 0.3–0.5 mm, abaxially flat, sessile, continuous with andropodium, adaxial surfaces each with 2 anther cells, (poorly preserved). Pollen unknown.

Gynoecium (including style-stigma) 2.5–3.5 mm by 2.2–3 mm; ovary erect, isolobous, ellipsoid, circular in transverse section 2–2.5 mm long, 1.7–1.9 mm diam.; bilocular, placentas hemi-ellipsoid, 1.4 x 0.7 x 0.4 mm, each with c. 90 ovules–seeds; outer surface of ovary-fruit with 14 longitudinal ridges, commissural ridges absent, ridges extending from base to apex of ovary, except the two pairs nearest to the commissure (valve junction) which are parallel to each other (straight not curved) and which extend only half the length of the ovary; ridges flat-topped, broader than long, c. 0.1 mm deep by 0.2–0.25 mm wide. Style-stigmas 2, bases connate into a funneliform cylinder, 1.5 mm wide at base, 2 mm wide at apex, deeply bifurcate almost to the base at the locule junction (commissure), apices abruptly reflexed, as a keel, encircling the perimeter of the distal half of the ovary-fruit, 0.5 mm high, persistent, papery, in the fruit.

Fruit septifragally dehiscent into two equal valves; valves persistent, separating only slightly. Seeds dry, oblong, c. 0.25 mm by 0.1 mm, outer epidermis mucilaginous, greatly swelling when hydrated (Fig 1).

#### Distribution

**Fig 2:**
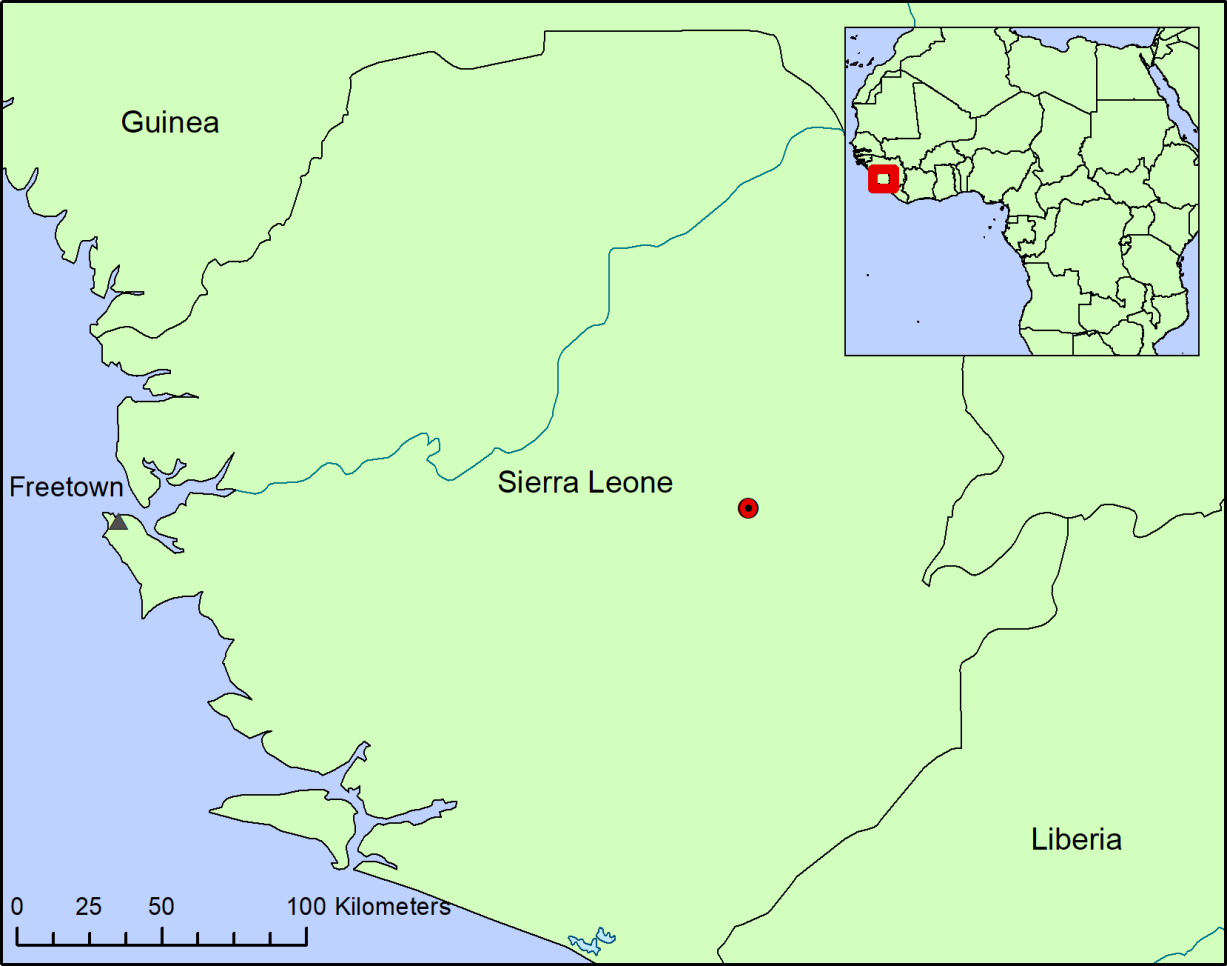
Map showing the global distribution of *Lebbiea grandiflora*. Based on the single herbarium record known, *Lebbie* A2721.

Known only from one site on the Sewa River in the Kono and Kenema districts of Eastern Sierra Leone. The nearest settlement is Nɡnawama, several kilometres drive from Jaiama Sewafe, a large settlement dependent on alluvial diamond mining (Fig 2).

#### Etymology

The generic name *Lebbiea* commemorates Dr. Aiah Lebbie, Head of The National Herbarium of Sierra Leone, Njala University, Sierra Leone who collected the type and only known material of this genus. The specific epithet refers to the flowers which are the largest known in the family in Africa (4-5 mm long above the pedicel), exceeding even those of *Dicraeanthus africanus* Engl. (3.5 mm long above the pedicel).

#### Conservation

Here *Lebbiea grandiflora* is assessed as Critically Endangered CR B1+B2ab(iii) according to the categories and criteria of IUCN [25]. This is because it is known from a single location in the Sewa River, at which there are two severe threats (see below). The area of occupancy is 1km^2^ using the IUCN-preferred cell-size for aquatic organisms. In fact the species is only known from three patches varying from 60 cm x 120 cm to 150 x 90 cm in size, all within a square measuring approximately 250m x 250m.

A total of five days search effort was spent targetting Podostemaceae on the Sewa River in April-May 2017, by a team of six personnel. Although numerous records of several Podostemaceae species were made along the length of the river, *Lebbiea grandiflora* was exceptional in being only recorded at this one location. Here it appeared to be greatly threatened. At this point the bed of the Sewa River is about 250m wide (measured on Google Earth), formed of flat rock, which is the substrate of Podostemaceae. However much of this substrate was becoming covered in particles of gravel and coarse sand that had been washed downstream from numerous alluvial gold and diamond mining operations on the riverbed and banks upstream (A. Lebbie pers. comm. Sept. 2017). These operations are clearly visible on Google Earth (viewed Sept. 2017) for many tens of kilometres upstream of the type locality. As more sediment is deposited on the only known habitat for *Lebbiea grandiflora*, so its opportunity for re-establishment each year from seed is reduced. Podostemaceae require a stable, solid surface on which to establish as seedlings. Alluvial diamond and gold extraction has already been implicated in the extinction of *Ledermanniella lunda* Cheek [5]. A second threat to *Lebbiea grandiflora* is the planned construction of a hydroelectric installation at the Bekongor Falls (on the Sewa River) some 3 km south of the type locality. Such installations pose a grave threat to Podostemaceae. For example, 19 of the 30 species of *Inversodicrea* are threatened with extinction due to such projects [7]. In 2018 it is intended to attempt to refind *Lebbiea grandiflora* in the wild so that seed can be collected to attempt ex situ conservation. However, successful establishment of Podostemaceae from seed at a new location has never been reported. Formal publication of this species in this paper is expected to facilitate the possibility of support for conservation efforts from the national authorities.

#### Ecology

*Lebbiea grandiflora* grows on submerged rocks in river beds, in the wet season, where they have been found in the middle of the river and close to the river bank. At the height of the dry season when falling water levels have exposed the rocks, the plants flower and fruit and die off (they can be easily scraped off the rock at this time). They grow in small isolated patches on rocks, covering the horizontal surfaces in a mat-like appearance with the flattened roots. Two of the observed clumps were growing on rocks in the middle of the river, with the third one close to the edge of the bank where a small rapid was still observable at the height of the dry season. The mass of rock on which it was growing had been partly dissected by the erosive forces of the river current, developing numerous small basins in which water was still present, sand and pebbles from the alluvial diamond mining had also settled. In the cracks in the bedrock and at the edges of the numerous basins can be found tufted water grasses growing in association with hydrophytes such as *Hygrophila spp*. (Acanthaceae). These plants probably serve to modulate the water current over the rocks in the wet season. *Pterocarpus santalinoides* L'Hér. ex DC. (Leguminosae-Papilionoideae) was the only tree growing on these rocks in the middle of the river.

## Discussion

### Subfamilial placement of *Lebbiea*

Due to the presence of broad tepal members that enfold the gynoecium, *Lebbie*a cannot be accommodated in Podostemoideae as currently circumscribed, since all species of that subfamily have highly reduced, more or less filamentous tepals. Both Weddellinoideae and Tristichoideae, the remaining subfamilies of Podostemaceae, share with *Lebbie*a broad tepals. This poses the question as to whether it might be accommodated in either of those two subfamilies.

*Lebbie*a shares with Weddellinoideae the 2-locular ovaries (3-locular in Tristichoideae). However, Weddellinoideae (and Tristichoideae) differ radically from *Lebbie*a in their lack of a spathellum, in their androecial structures, tepal number and capsule valve rib number (Table 1). These features have also traditionally been used in delimiting the subfamilies of Podostemaceae [8].

**Table 1:** Comparison of *Lebbiea* with the three subfamilies of Podostemaceae. Characters for subfamilies taken from [8].

	Tristichoideae	Weddellinoideae	*Lebbiea*	Podostemoideae
Perianth lobes	Ovate-elliptic	Ovate-elliptic	Ovate	Filiform
Spathellum	Absent	Absent	Present	Present
Tepal number	3	(4-)5(-6)	2	2-many
Stamen number and attachment	1-3, free	5-25, free	3, united on andropodium	1-many, often united on andropodium
Carpel number	3	2	2	2
Capsule valve rib number	3	3	7	0-many

However, identical or similar character states to those listed above do occur in the Podostemoideae. There is a choice:

1. Erect a new subfamily for *Lebbie*a. This option is supported by the possession of three traits apparently unique in the Podostemaceae:
  - andropodium highly differentiated: with robust, mechanically strong, self-supporting capacity, having differentiated thickened central, and angled, thinner marginal areas. In other Podostemaceae the andropodial structures are undifferentiated. If andropodia are present (e.g. most *Inversodicraea*) they appear terete, and to be formed only by simple connation of staminal filaments, and like those, are supported only by hydrostatic pressure. Consequently they wilt if the plant becomes dehydrated (Cheek pers. comm. 2017 in relation to African Podostemaceae). However non-terete, flattened andropodia do appear to occur in *Podostemum* e.g. *P*. *distichum* (Chamisso) Weddell [30].
  - a highly developed and robust style-stigma structure in which the bases of the two style-stigmas unite to form a cleft funneliform-cylindrical structure, with a reflexed, blade-like apex that extends half-way around the perimeter of the ovary-fruit towards the base of the ovary-fruit.
  - highly developed, pillar-like haptera that completely elevate the crustose root above the substrate. Although haptera are well-known in Podostemaceae, they are usually lateral to the root which remains appressed (and usually due to root-hairs adhering) to the substrate. This trait may serve to give the plant an advantage over co-occurring Podostemacae in its habitat, allowing it to grow-over and shade-out competitors. A reviewer has speculated that these pillar-like haptera may be artefacts of death, but the authors do not support this view.
2. Inclusion of *Lebbie*a in a recircumscribed Podostemoideae. This would broaden the delimitation of Podostemoideae so as to remove one of the key morphological points of distinction between Podostemoideae and Tristichoideae and Weddellinoideae, that is, the absence of foliose tepals in Podostemoideae versus their prescence in the last two subfamilies

We opt to include *Lebbie*a within an expanded concept of Podostemoideae as a distinct new genus that necessitates a broader morphological delimitation of the subfamily. We hope that DNA might become available, allowing a molecular phylogenetic analysis that will test this hypothesis. However, given the precarious survival of this plant in the wild, this may never be possible.

### Placement of *Lebbiea* within Podostemoideae

If *Lebbiea* is placed in Podostemoideae, its morphology suggests that, with three other West African species, it may be sister to most African podostemoids. This is because it has a 2-locular ovary, known in only two small groupings of African podostemoids which are otherwise unilocular. One of the bilocular groups is the three Ghanaian species attributed to the genus *Saxicolella* Engl. These Ghanaian species are sister to all other Old World (and some New World) Podostemoids in the molecular phylogenetic analysis of Koi et al. [2]. Bilocular ovaries also occur in the predominantly Guinean-Sierra Leone genus *Stonesia* G.Taylor, but *Stonesia* was not sampled for the Koi et al. study, apart from the anomalous unilocular Cameroonian species *Stonesia ghoguei* E.Pfeifer & Rutish. [31], which was placed in the main crown clade of African podostemoids [3]. This species is conjectured here to be misplaced in *Stonesia*.

The broad, functional (protective) tepals of *Lebbiea* are also concordant with a basal position in African Podostemoids since broad lobes are plesiomorphic with the basal subfamilies Tristichoideae and Weddellinoideae.

Among the African podostemoids, apart from sharing a bilocular ovary, *Lebbiea* shares three unusual character states with *Stonesia*. These are a) the presence of a pair of near-commissural ribs (not placed on the commissure itself as is usual in African podostemoid genera, but set back from it) which are further unusual in that they do not extend from ovary apex to base; b) the number of longitudinal fruit ribs is high (15). In *Stonesia*, the range is 12-16 [16]. In all other African genera of podostemoids, such ribs if present do not exceed 8 [21]; c) a long andropodium which bears 3 anthers at the top (although in *Stonesia* the central anther is a filamentous staminode, and the fertile anthers are set on free filaments, not sessile).

Therefore, we conjecture that a sister relationship between *Stonesia* and *Lebbiea* is most credible. However, since *Stonesia* sensu stricto has not been placed in any phylogenetic analysis, this is not very informative. The two genera differ greatly in the features given in the diagnosis below and in Table 2.

**Table 2:** Characters separating *Lebbiea* from *Stonesia* (*Stonesia* characters taken from [16] and pers. obs. Cheek 2018).

	*Stonesia*	*Lebbiea*
Haptera	Absent	Well-developed
Leaves	Deeply lobed to repeatedly bifurcate	Entire
Tepals	Filiform	Ovate, foliose
Andropodium	Terete, simple structure, posture maintained by hydrostatic pressure (wilts)	Flattened, posture maintained by complex structure (non-wilting)
Anthers	Borne on short free filaments from andropodium	Sessile on andropodium
Anther number on andropodium	Central staminode flanked by 2 anthers	3 equal anthers, staminode absent
Style-stigma base	Style-stigma base free, botuliform to filiform	Style-stigmas conjoined at base forming bifurcate funneliform cylinder
Style-stigma apex	Not distinct from style –stigma base, erect	Reflexed, keel-like, encircling distal part of ovary.
Near-commissural ribs of undehisced fruit	Curved	Straight

### New taxon discovery in Sierra Leone

*Lebbiea* is the second new genus of angiosperm discovered from Sierra Leone this century, following the publication of *Karima* Cheek & Riina in 2016 [24], also a result of a baseline study for an Environmental Impact Assessment (EIA) for a hydro-electric project. The number of new species discovered in this time-frame is larger. Examples of such newly discovered species in Sierra Leone in alphabetical order by genus, together with their conservation assessment (from the protologue or http://www.redlist.org) are: *Dactyladenia globosa* Jongkind [32] (Endangered), *Eriocaulon cryptocephalum* S. M. Phillips & Mesterházy and *E*. *tingilomum* S. M. Phillips & Mesterházy [33] (not assessed), *E*. *sulanum* S. M. Phillips & Burgt and *E*. *petraeum* S. M. Phillips & Burgt [34] (both Critically Endangered), *Gilbertiodendron tonkolili* Burgt & Estrella [35] (Critically Endangered), *Isoglossa dispersa* I. Darbysh. & L. J. Pearce [36] (Vulnerable), *Leptoderris sassandrensis* Jongkind [37] (not assessed), *Napoleonaea alata* Jongkind [38] (Vulnerable), *Pseudovigna sulaensis* R. Clark & Burgt [39] (Vulnerable), *Psychotria samoritourei* Cheek [40] (Vulnerable), *Stylochaeton pilosus* Bogner [41] (Endangered), and *Xysmalobium samouritourei* Goyder [42] (Endangered). Just over the border in Guinea examples include *Gymnosiphon samoritoureanus* Cheek [43] (Vulnerable), *Striga magnibracteata* Eb. Fisch. & I. Darbysh. [44] (Endangered) and *Kindia gangan* Cheek [45] (Endangered).

These species illustrate three points: 1) that even in tropical African countries considered well-sampled such as Sierra-Leone, numerous new species and even genera still remain to be discovered while natural habitat persists; 2) like *Lebbiea*, available data for most of these species shows that they are range-restricted or rare in habitat that is threatened, and so, when assessed for their conservation status, threatened with extinction; 3) the majority of these discoveries (9/16: all excepting [32, 33, 36,37,41 and 45]) have resulted from (high quality) EIA related studies due to mining and infrastructure projects. That EIA studies make such a major contribution to current species discovery points to the scarcity of resources from other sponsors to support botanical inventory at a time when species are probably being lost before they are discovered.
